# Real-time ultrasound-guided subclavian vein cannulation in cardiac surgery: comparison between short-axis and long-axis techniques

**DOI:** 10.1186/cc13322

**Published:** 2014-03-17

**Authors:** F Corradi, T Manca, C Brusasco, F Cocconcelli, A Agostinelli, F Benassi, T Gherli, A Vezzani

**Affiliations:** 1 Ente Ospedaliero Ospedali Galliera Genova, Italy; 2 Azienda Ospedaliero- Universitaria di Parma, Italy; 3 Università degli Studi di Genova, Italy

## Introduction

Central venous catheters play an important role in patient care; however, their use is associated with various complications and more frequently through the subclavian vein (SCV) route. A previous study showed that ultrasound-guided cannulation of the SCV in critical care patients is superior to the landmark method and should be the method of choice in these patients [1]. The aim of this study was to compare short-axis and long-axis approaches for ultrasound-guided subclavian vein cannulation with respect to indicators of success.

## Methods

Eighty-three patients undergoing cardiac surgery and requiring central venous cannulation were randomized to receive long- axis or short-axis ultrasound-guided cannulation of the subclavian vein by a skilled anesthesiologist. First-pass success, unsuccessful placement, number of attempts, number of needle passes, skin and vessel puncture, time to successful catheterization and complications were considered as outcomes.

## Results

The subclavian vein was successfully cannulated by ultrasound- guided techniques in all patients. Central venous cannulation failed in two and 10 cases respectively with short-axis and long-axis view and the other view was used successfully. The first-pass success rate was significantly higher in the short-axis group (73%) compared with the long-axis group (40%) (*P *= 0.005). The procedure time, number of attempts, needle redirection, and skin and vessel punctures were significantly lower in the short-axis than long-axis group (*P *< 0.05). The overall number of complications did not differ significantly between groups even if artery puncture and hematoma occurred more frequently in the long-axis group. Moreover, the need to change the ultrasound- guided insertion technique was more frequent in the long-axis group.

## Conclusion

Ultrasound-guided subclavian vein cannulation by an experienced operator has a higher first-pass success rate and lower access time using the short-axis than long-axis approach.
